# Anthropometrics and Body Composition by Dual Energy X-Ray in Children of Obese Women: A Follow-Up of a Randomized Controlled Trial (the Lifestyle in Pregnancy and Offspring [LiPO] Study)

**DOI:** 10.1371/journal.pone.0089590

**Published:** 2014-02-24

**Authors:** Mette Tanvig, Christina A. Vinter, Jan S. Jørgensen, Sonja Wehberg, Per G. Ovesen, Ronald F. Lamont, Henning Beck-Nielsen, Henrik T. Christesen, Dorte M. Jensen

**Affiliations:** 1 Department of Endocrinology, Odense University Hospital, Odense, Denmark; 2 Department of Gynecology and Obstetrics, Odense University Hospital, Odense, Denmark; 3 Institute of Clinical Research, University of Southern Denmark, Odense, Denmark; 4 Centre for Clinical Epidemiology, Odense University Hospital, Odense, Denmark; 5 Department of Gynecology and Obstetrics, Aarhus University Hospital, Skejby, Denmark; 6 Division of Surgery, University College London, London, United Kingdom; 7 Hans Christian Andersen Childreńs Hospital, Odense University Hospital, Odense, Denmark; Kyushu University Faculty of Medical Science, Japan

## Abstract

**Objective:**

In obese women, 1) to assess whether lower gestational weight gain (GWG) during pregnancy in the lifestyle intervention group of a randomized controlled trial (RCT) resulted in differences in offspring anthropometrics and body composition, and 2) to compare offspring outcomes to a reference group of children born to women with a normal Body Mass Index (BMI).

**Research design and methods:**

The LiPO (Lifestyle in Pregnancy and Offspring) study was an offspring follow-up of a RCT with 360 obese pregnant women with a lifestyle intervention during pregnancy including dietary advice, coaching and exercise. The trial was completed by 301 women who were eligible for follow-up. In addition, to the children from the RCT, a group of children born to women with a normal BMI were included as a reference group. At 2.8 (range 2.5–3.2) years, anthropometrics were measured in 157 children of the RCT mothers and in 97 reference group children with Body Mass Index (BMI) Z-score as a primary outcome. Body composition was estimated by Dual Energy X-ray (DEXA) in 123 successful scans out of 147 (84%).

**Results:**

No differences between randomized groups were seen in mean (95% C.I.) BMI Z-score (intervention group 0.06 [−0.17; 0.29] *vs.* controls −0.18 [−0.43; 0.05]), in the percentage of overweight or obese children (10.9% *vs.* 6.7%), in other anthropometrics, or in body composition values by DEXA. Outcomes between children from the RCT and the reference group children were not significantly different.

**Conclusions:**

The RCT with lifestyle intervention in obese pregnant women did not result in any detectable effect on offspring anthropometrics or body composition by DEXA at 2.8 years of age. This may reflect the limited difference in GWG between intervention and control groups. Offspring of obese mothers from the RCT were comparable to offspring of mothers with a normal BMI.

**Trial registration:**

clinicaltrials.gov NCT00530439, NCT01918319 and NCT01918423. URL: NCT00530439&quest;term&hairsp;&equals;&hairsp;NCT00530439&amp;rank&hairsp;&equals;&hairsp;1, NCT01918319&quest;term&hairsp;&equals;&hairsp;NCT00530439&amp;rank&hairsp;&equals;&hairsp;2 and NCT01918423&quest;term&hairsp;&equals;&hairsp;NCT00530439&amp;rank&hairsp;&equals;&hairsp;3.

## Introduction

The prevalence of overweight and obese children has increased in recent decades worldwide [1]. Over two thirds of obese children become obese adults [2], [3] and maternal obesity is linked to obesity of their offspring in both early [4]–[6] and later life [7], [8]. Other maternal factors such as impaired glucose tolerance and excess gestational weight gain (GWG) are also associated with adverse effects on the body composition of offspring [9]–[12]. The underlying mechanisms are poorly understood, but an unfavorable intrauterine environment in which mother and fetus share excess nutrients has been suggested [13]–[15]. This intergenerational cycle of obesity cannot be explained by genetics alone, since differences in birth weight between siblings according to maternal GWG and differences in overweight and obesity rates in siblings born before and after maternal substantial weight loss have been observed [16], [17]. Once present, obesity is difficult to treat and early intervention strategies are urgently needed. Pregnancy offers the opportunity to manage or prevent obesity in both mother and child, and though a number of intervention studies involving overweight or obese pregnant women have reported on maternal and perinatal outcomes [18], follow-up data in the offspring have not been reported. The Danish Lifestyle in Pregnancy (LiP) study was a randomized controlled trial (RCT) with lifestyle intervention in obese pregnant women [19]. The aim of the present study (Lifestyle in Pregnancy and Offspring, LiPO) was to assess the effect of this pregnancy intervention on the body composition of the offspring (at 2.5–3 years of age) of women recruited to the LiP study and to compare outcomes with children born to women with normal weight. In the LiP study, participants in the intervention group had a significantly lower GWG compared to controls [19], and our hypothesis was that this would result in a lower body mass index (BMI) and a healthier body composition in the offspring of these women.

## Materials and Methods

The protocol for this trial and supporting CONSORT checklist are available as supporting information; see Checklist S1 and Protocol S1.

### Ethics statement

Both the LiP and the LiPO study were approved by the local ethics committee of the Region of Southern Denmark and the Danish Data Protection Agency. The LiP study was registered at www.clinicaltrials.gov as NCT00530439. The LiPO follow-up was planned in 2010, while the LiP study was still running, and was registered at www.clinicaltrials.gov as NCT01918319 for comparison of offspring of mothers participating in the LiP study, and as NCT01918423 for comparison of offspring from the LiP study with the reference group of children born to normal weight women. Written informed consent was obtained for each participant, initially as part of the LiP study and again for participants of the LiPO follow-up. All aspects of both studies were conducted according to the Declaration of Helsinki.

### Data collection and handling

The LiP study was a RCT that was conducted in Odense University Hospital and Aarhus University Hospital, Denmark between October 2007 and October 2010. A total of 360 women aged 18–40 years were recruited at 10–14 weeks of gestation. The inclusion criterion was a BMI of 30–45 kg/m^2^ based on prepregnancy weight or first measured weight in pregnancy. Participants were randomized in a ratio of 1∶1 to: i) lifestyle intervention including dietary advice, coaching and exercise or to ii) routine obstetric care. Randomization was carried out using computer-generated numbers in closed envelopes. Subsequently, there was no blinding to patients or healthcare professionals. The lifestyle intervention in pregnancy consisted of two major components: i) dietary counseling and ii) physical activity. Trained dieticians carried out individual dietary counseling four times during pregnancy. The counseling was based on the evaluation of each participants dietary history, weight and level of activity and led to a personalized diet. The physical activity component consisted of encouragement to be moderately physically active for 30–60 minutes daily. Each participant was given free, full-time membership in a fitness center, where they could choose between several different types of aerobic classes or weight training. Additionally, for one hour each week, a closed aerobics class was arranged with a physiotherapist, and participants were requested to attend this session [19]. Women in both groups were monitored with fasting blood samples, oral glucose tolerance tests (OGTTs) and weight measurement. The methods are described in detail in a prior publication [19]. The intervention group had a significantly lower median GWG compared with the control group (7.0 *vs*. 8.6 kg; *p* = 0.01). No significant differences were seen in the five main clinical outcomes between groups (gestational diabetes, preeclampsia/pregnancy induced hypertension, cesarean delivery, infants born large for gestational age or infants admitted to neonatal intensive care unit) [19]. As part of the LiP study, women were seen six months post partum, where breastfeeding information was gathered. For the present study, we additionally included a reference group of children born to women with a normal BMI. These mother and child dyads were identified from electronic patient records. The inclusion criteria for the reference group were: singleton children born at term in Odense University Hospital from September 2008 to September 2009 to normal weight (prepregnancy BMI 18.5–24.9 kg/m^2^), healthy Caucasian women. Exclusion criteria for the reference group were: children born before 37 or after 41 completed weeks of gestation, children with significant medical conditions (defined by being hospitalized for more than 10 days in the first year of life), and maternal serious obstetric complications, pre-existing or gestational diabetes, hypertensive disorders or mental illness.

**Figure 1:**
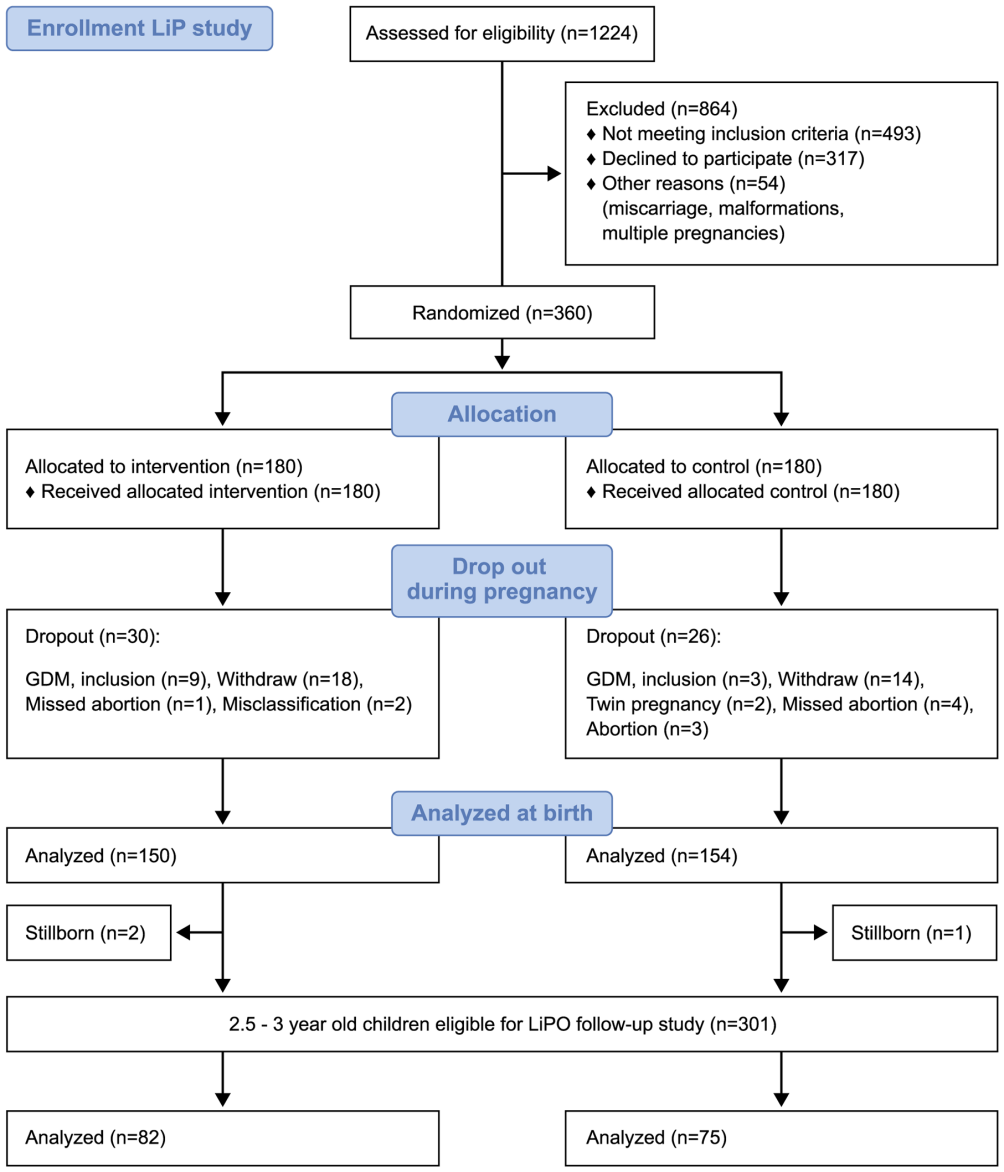
Participation rates in the LiP and LiPO studies. Legend: GDM  =  gestational diabetes mellitus. LiP  =  Lifestyle in Pregnancy. LiPO  =  Lifestyle in Pregnancy and Offspring.

Of the initial 360 included women in the LiP study, 304 participated in the trial until birth (Figure 1). At delivery, three children were stillborn (two in the intervention group and one in the control group). Accordingly, 301 mother and child dyads were eligible for the LiPO infant follow-up study. For the reference group, we identified 325 eligible mother and child dyads. Trained midwives measured all children at birth according to national guidelines. Maternal baseline data were obtained from the LiP study and included GWG (estimated from weight measured at gestational week 35 minus weight at study inclusion), maternal fasting and 2-h glucose levels on OGTT carried out at 28 weeks gestation, maternal age, educational level, employment and smoking during pregnancy. For the reference group, maternal baseline data were obtained from electronic patient records (prepregnancy BMI, parity and maternal age), from postal questionnaires 24 months post partum (smoking during pregnancy, educational level and employment) or at follow-up exams (self-reported GWG). We had no information on glucose levels during pregnancy in mothers from the reference group, as this was not measured. Information on breastfeeding patterns for LiP study participants was obtained at follow-up visits six months post partum as part of the LiP study and from postal questionnaires 12 months post partum. Breastfeeding data for the reference group were obtained from postal questionnaires 24 months post partum. At age 2.5 years, all eligible participants were invited to attend for clinical examination. Non-responders were contacted twice in order to improve the participation rate. Follow-up visits between the age of 2.5 and 3 years were conducted at Odense University Hospital or Aarhus University Hospital between February 2011 and November 2012. A Dual-Energy X-ray absorption (DEXA) scan for assessment of body composition was performed in Odense University Hospital. One doctor (M.T.), who was blinded to the LiP intervention, examined all the children and un-blinding only occurred after data collection was complete. Due to identifiable differences in maternal BMI, it was not possible to blind M.T. to the reference group. After the clinical examination, the pediatric medical records were reviewed and data on weight and height at 5 and 12 months of age were collected from routine visits to general practitioners.

### Anthropometry

Weight in light indoor clothing was measured to the nearest 0.1 kg using a digital weight (model 704, Seca, Hamburg, Germany). Height was measured to the nearest 0.1 cm using a portable stadiometer (model 214, Seca, Hamburg, Germany). Triceps and subscapular skinfold thickness was measured to the nearest 0.1 mm using a Harpenden skinfold caliper (Chasmors Ltd, London, UK). Abdominal circumference at the level of the umbilicus and hip circumference at the widest diameter of the buttocks were measured to the nearest mm with a non-stretchable tape measure. All measures were performed in triplicate and averaged.

### DEXA scans

On the same day as the anthropometric evaluation, a trained research bioanalyst and M.T. performed and evaluated the DEXA scans using a Lunar Prodigy scanner (GE Healthcare, Madison, WI, USA) that was equipped with the ENCORE software (version 12.3, Prodigy; Lunar Corp, Madison, WI, USA). Approximately 45 minutes were allocated for each child. Due to the young age of the children, the quality of the DEXA scans varied and some were inadequate. Consequently, scans were categorized as previously suggested [20]: i) perfect, ii) good with minor irregularities, iii) several irregularities, iv) unusable. Scans graded iii) or iv) were excluded from further analyses.

### Outcomes

The primary outcome was the child BMI Z-score (standard deviation score). BMI was calculated as weight (kg) divided by the square of height (m^2^) and expressed as a continuous Z-score based on age and sex-specific Danish standards [21]. Secondary outcomes were BMI, triceps skinfold thickness, mid-scapular skinfold thickness, abdominal circumference, hip circumference, abdominal/hip circumference ratio, and the DEXA values of total fat mass, total lean mass and fat percentage. Furthermore, overweight or obese children were identified using the criteria defined by the International Obesity Task Force (IOTF) Childhood Obesity Working Group [22]. As few children were identified as being obese, we chose to classify overweight and obese children as one outcome.

### Statistical analyses

All analyses were performed using STATA 12.0 software (StataCorp, College Station, TX), and in all analyses, a significance level of 0.05 (two-sided) was chosen. With no previous studies available on which to base a power calculation, we aimed to include 160 of the 301 eligible children from the LiP study (53%). Given an alpha of 0.05, a beta of 0.80 and a BMI Z-score SD of 1.0, a true difference between the LiP intervention and the control group in offspring BMI Z-score of 0.447 could be detected. In order to have a sufficient reference group, we aimed to include a minimum of 90 children born to women with a normal BMI. Baseline differences between the groups and those participants lost to follow-up from the LiP study were analyzed first with One-way Anova, Kruskal-Wallis or where appropriate, Chi^2^ test. Subsequently, the analyses were done; i) between randomized groups attending the follow-up and ii) between all those attending the follow-up and those lost to follow-up, using Student's t-test, Mann-Whitney U test or Chi^2^ test, where appropriate. We did not perform statistical testing for baseline differences between randomized groups and the reference group as the latter was selected from a different population (pregnant women with a normal BMI). Outcome differences between the randomized groups from the LiP study were analyzed initially using the Student's t-test, Mann-Whitney U test or Chi^2^ test. Subsequently, outcome differences between all three groups (intervention, control and reference) were analyzed with One-way Anova, Kruskal-Wallis or Chi^2^ test. Since children born preterm (<37 completed weeks of gestation) have different growth patterns than children born at term [23], we made two series of analyses, one with all children included, and one including only healthy children (no serious medical conditions) born at term.

As the reference group was not part of the LiP study, potential confounders might have contributed to differences in outcomes. Accordingly, we also performed a simple followed by a multiple linear regression analysis to estimate differences in our primary outcome (BMI Z-score) between the three groups, adjusting for potential confounders in the multiple regression. Potential confounders included maternal age, parity, educational level (school ≥12 years), GWG, smoking during pregnancy, breastfeeding (exclusive breastfeeding for at least 5 months), birth weight Z-score and post natal excessive growth. Birth weight Z-scores were calculated from recently published gestational age and sex-specific Danish birth weight standards [4], and excessive growth was estimated by investigating changes in weight Z-scores (calculated from the current Danish reference [21]) from 0 to 12 months. Only children with information on all the above variables were included, and since we did not have information on glucose values during pregnancy in the reference group, this variable was not included in the analysis. Children born preterm (<37 completed weeks of gestation) and children with severe medical conditions were not included in the regression analyses.

## Results

### Participants and baseline characteristics

Of the 301 eligible LiP study and 325 reference group mother and child dyads, 157 (52.2%) and 97 (29.8%) respectively were seen for the LiPO follow-up (Figure 1). Of the 325 eligible reference group mother and child dyads, 97 (29.8%) were seen. All participants were of Caucasian race. Most of the eligible mothers were employed and the main reason for declining to participate in the follow-up was lack of time. Overall, participants from the intervention (n = 82) and control group (n = 75) did not differ with respect to maternal or neonatal baseline characteristics (Table 1). At baseline, there were no differences between those who attended and those who were lost to follow-up except for 2-h OGTT plasma glucose values performed at 28 weeks gestation (Table 1). Compared to women from the LiP study, those from the reference group had a lower BMI, higher educational level and higher GWG. Children from the reference group had a lower mean birth weight, whereas there were no differences in abdominal circumference or length at birth.

**Table 1:** Baseline, pregnancy and neonatal outcome data in trial groups from the LiP study and from a reference group of children born to women of normal weight.

	Participants in the LiP study			Reference group	
	Intervention	Control	Lost to follow-up	Children born to women of normal weight	Missing numbers (intervention/controls/lost to follow-up/reference)
	n = 82	n = 75	n = 144	n = 97	
*Maternal*					
Age at delivery (years)	30.5 (29.6; 31.3)	30.0 (29.0; 31.0)	30.1 (29.4; 30.8)	30.2 (29.4; 31.1)	.
Primiparous	42 (51.2%)	42 (56.0%)	74 (51.4%)	41 (47.7%)	.
Prepregnancy BMI (kg/m^2^)	34.1 (33.4; 34.8)	34.3 (33.6; 35.0)	34.4 (33.9; 35.0)	22.0 (21.7; 22.4)	.
Prepregnancy BMI 30–34.9 (kg/m^2^)	54 (65.8%)	47 (62.7%)	93 (64.5%)	.	.
Prepregnancy BMI 35–39.9 (kg/m^2^)	20 (24.4%)	25 (33.3%)	42 (29.2%)	.	.
Prepregnancy BMI 40–45 (kg/m^2^)	8 (9.8%)	3 (4.0%)	9 (6.3%)	.	.
Smoking in pregnancy	4 (4.9%)	7 (9.3%)	17 (11.8%)	10 (10.5%)	.
School ≥12 years	62 (75.6%)	48 (64.0%)	96 (66.7%)	97 (100.0%)	.
Further education ≥3 years	42 (51.2%)	33 (44.0%)	66 (45.8%)	85 (87.6%)	.
Employed in work	54 (65.9%)	55 (76.4%)	97 (67.4%)	78 (80.4)	.
Gestational weight gain	7.7 (6.8; 8.7)	8.8 (7.7; 9.8)	7.7 (6.9; 8.5)	15.9 (14.8; 17.0)	5/2/6/2
75-g OGTT at 28 weeks of gestation:					
-Fasting plasma glucose (mmol/L)	4.90 (4.8; 5.0)	4.90 (4.8; 5.0)	4.9 (4.8; 5.0)	.	8/1/19/97
-2-h plasma glucose (mmol/L)	6.3 (6.0;6.6)	6.2 (5.9; 6.5)	6.7 (6.4; 6.9)	.	12/5/27/97
*Neonatal*					
Sex, female/male	41/41	33/42	66/78	47/50	.
Gestational age at birth (days)	279 (275; 283)	281 (278;283)	281 (278; 283)	281 (279; 283)	.
GA <37+0	5 (6.2%)	2 (2.3%)	6 (4.2%)	.	.
Birth weight (g)	3634 (3479; 3788)	3616(3505; 3727)	3685 (3585; 3786)	3555 (3467; 3641)	.
Birth weight >4000g	23 (28.7%)	16 (21.3%)	39 (27.0%)	20 (20.1%)	.
Large for Gestational Age	11 (13.4%)	8 (10.7%)	21 (14.5%)	6 (6.2%)	.
Birth weight Z-score	0.33 (0.10; 0.57)	0.11 (−0.13; 0.36)	0.35 (0.12; 0.58)	−0.07 (−0.26; 0.11)	.
Birth AC (cm)	33.6 (32.9; 34.3)	33.5 (33.1; 34.0)	34.0 (33.7; 34.4)	33.7 (33.3; 34.0)	.
Birth length (cm)	52.2 (51.4; 52.9)	52.4 (51.9; 52.9)	52.5 (52.1; 52.9)	52.7 (52.3; 53.2)	.

Legend: Data are given as mean and 95% C.I. or frequency. Differences between LiP randomized groups and lost to follow-up group were analyzed first with One-way Anova, Kruskal-Wallis or where appropriate, Chi^2^ test. Subsequently, the analysis was done 1) between randomized groups attending the follow-up and 2) between all attending the follow-up and those lost to follow-up, using Student's t-test, Mann-Whitney U test or Chi^2^ test, where appropriate. At a significance level of 0.05 (two-sided), no differences between randomized groups were detected and the only statistically significant difference between the intervention, control and lost to follow-up groups from the LiP study was 2-h plasma glucose values from the OGTT. IOM; Institute Of Medicine, LiP; Lifestyle in Pregnancy, OGTT; oral glucose tolerance test, GA; gestational age, AC; abdominal circumference.

### Follow up 0–3 years

Of the 250 children for whom breastfeeding data were available, 124 (49.6%) were exclusively breastfed (never formula fed) and 66 (26.4%) were exclusively breastfed for at least 5 months, with no differences between the intervention groups, or between intervention groups and reference group. Data on weight development from 0–5 months and from 0–12 months were obtained in 218 children. There was no difference in weight development between the intervention groups, or between intervention groups and reference group (data not shown). Among the children born preterm, two children from the intervention group and one child from the control group had severe medical conditions. None of the children born at term had severe medical conditions.

### Outcomes

Follow up was performed at a mean age of 1031 days (range 918–1155 days) equivalent to 2.8 years (range 2.5–3.2 years). Anthropometric measures and DEXA scan results are presented in Table 2. No differences were seen in mean (95% C.I.) BMI Z-score in children from the intervention group *vs.* controls (0.06 (−0.17; 0.29) *vs.* −0.18 (−0.43; 0.05)), nor were there any statistically significant differences in BMI Z-score between the LiP offspring and the reference group (−0.21 (−0.38; −0.04)), estimated by Oneway Anova. In the linear regression models which analyzed differences in BMI Z-score between the three groups, intervention group children had a non-significant trend towards a higher BMI Z-score (coefficient 0.27, *p* = 0.069 [crude values]) compared to the reference group, but this was not seen after adjusting for potential confounders (Table 3). Post regression model checking indicated that the multiple linear regression model was suitable. No differences between intervention, control or reference group were seen for the secondary outcomes: BMI (16.4 *vs.* 16.1 *vs.* 16.0 kg/m^2^), the percentage of overweight or obesity (10.9 *vs.* 6.7 *vs.* 4.1%), or for weight, length, skinfold thicknesses, abdominal circumference, hip circumference or abdomen to hip ratio (Table 2). DEXA scanning was successful in 123 (83.7%) of 147 children (intervention n = 37, controls n = 30, reference n = 56) at a mean age of 1035 days (2.84 years). No differences were detected in total fat mass (2.5 *vs.* 2.4 *vs.* 2.3 kg), total lean mass (11.3 *vs*. 11.2 *vs.* 10.9 kg) or fat percentage (21.6 *vs.* 21.6 *vs.* 21.3%) (Table 2). Similarly, comparative analyses of healthy children born at term showed negative results for all parameters (data not shown).

**Table 2:** Anthropometric outcomes and body composition according to LiP intervention and reference groups in 2.8

	LiP Offspring		Reference group
	Intervention	Control	Children born to women of normal weight
	n = 82	n = 75	n = 97
Age at exam (days)	1030 (1022; 1038)	1032 (1024; 1040)	1041 (1035; 1047)
BMI Z-score	0.06 (−0.17; 0.29)	−0.18 (−0.43; 0.05)	−0.21 (−0.38; −0.04)
Weight (kg)	14.7 (14.3; 15.1)	14.4 (14.1; 14.8)	14.4 (14.1; 14.7)
Height (cm)	94.6 (93.8; 95.3)	94.6 (93.8; 95.4)	94.7 (94.0; 95.3)
BMI (kg/m^2^)	16.4 (16.1; 16.7)	16.1 (15.8; 16.4)	16.0 (15.8; 16.2)
Overweight or obese	9 (10.9%)	5 (6.7%)	4 (4.1%)
AC (cm)	48.5 (47.9; 49.2)	47.9 (47.1; 48.7)	48.2 (47.6; 48.7)
Hip (cm)	50.8 (50.1; 51.5)	50.2 (49.4; 51.0)	50.4 (49.8; 51.0)
AC/hip ratio	0.97 (0.95; 0.97)	0.96 (0.95; 0.97)	0.96 (0.94; 0.96)
Triceps skinfold thickness (mm)	8.3 (7.9; 8.7)	8.3 (7.8; 8.8)	8.2 (7.8; 8.5)
Subscapular skinfold thickness (mm)	6.1(5.78; 6.52)	6.0 (5.7; 6.23)	5.8 (5.6; 6.1)
*DEXA scan*	n = 37	n = 30	n = 56
Total fat (g)	2463 (2147; 2779)	2442 (2189; 2696)	2325 (2117; 2532)
Lean body mass (g)	11 336 (10 942; 11 730)	11 236 (10 797; 11 675)	10 914 (10 617; 11 211)
Total fat (%)	21.6 (19.1; 24.1)	21.6 (19.7; 23.6)	21.3 (19.5; 23.1)

Legend: Data are given as mean and 95% C.I. or frequency. Differences between LiP offspring groups were analyzed with Student's t-test, Mann-Whitney U test or Chi^2^ test, where appropriate. Differences between LiP offspring and reference groups were analyzed with Oneway Anova, Kruskal Wallis or Chi^2^ test, where appropriate. At a significance level of 0.05 (two-sided), there were no statistically significant differences in any variables between the LiP intervention and control groups or between LiP groups and reference group. AC; abdominal circumference, DEXA; Dual Energy X-ray.

**Table 3:** Simple and multiple regression analyses showing crude and adjusted difference in BMI Z-score in the three groups of term children at age 2.8 years.

	Crude (n = 247)			Adjusted* (n = 192)		
	BMI Z-score Coefficient (95% C.I.)	*P*	R^2^	BMI Z-score Coefficient (95% C.I.)	*P*	R^2^
			0.02			0.35
Reference	Ref.			Ref.		
LiP Intervention	0.27 (−0.02; 0.56)	0.069		0.28 (−0.11; 0.67)	0.159	
LiP Control	0.03 (−0.27; 0.33)	0.858		0.18 (−0.22; 0.57)	0.384	

Legend: *Adjusted for gestational weight gain, parity, smoking during pregnancy, maternal age, educational level (school ≥12 years), breastfeeding (exclusive breastfeeding for at least 5 months), birth weight Z-score and excessive post natal growth (change in weight Z-score between 0 and 12 months). Only term children with available data on all variables were included in the adjusted analysis. Z-score; standard deviation score, LiP; Lifestyle in Pregnancy.

## Discussion

To our knowledge, this is the first infant follow-up from a large RCT [19] with lifestyle intervention for obesity in pregnancy. We were unable to detect any effect of our LiP intervention on offspring BMI Z-score, BMI or any other anthropometric measure. Similarly, no adverse outcomes were detected. For a subgroup of children, we assessed body composition by DEXA scans which are considered the “gold standard” for body composition analysis, but still detected no differences between intervention and control groups. In addition, no outcome differences were found between the offspring of obese pregnant women participating in the LiP study and a reference group of children born to women of normal weight. The negative results of the RCT on offspring anthropometry may reflect the limited difference in the effect of the lifestyle intervention in the pregnant mothers in the LiP study compared to controls [19] which became evident after this study was initiated. Even though a significant difference was found in GWG in the LiP study, due to small numbers, this difference was no longer significant. Furthermore, compared to similar intervention studies, participants in the LiP study had a low GWG in both randomized groups [24]. Previous studies have suggested that only weight gain in early pregnancy is associated with increased offspring adiposity [25], [26] so it may also be the timing of intervention in the LiP study with inclusion of women between 10 and 14 weeks of gestation that prevented us from to detecting differences between the groups. We were able to achieve an attendance rate of 52.2% in our follow-up of the LiP study, and though this is comparable to similar follow-up studies [27], [28], it may have limited the validity of our findings. We almost met our aim of 160 LiP follow-ups, but the power calculation used to calculate this number was based on the expectation of a large difference in BMI Z-score (1SD), which in hindsight probably was unrealistic given the small difference in GWG in the LiP study. However, as the LiPO follow-up was planned while the LiP study was still running, and as we had no previous studies on which to base power calculation, we had no better option. Accordingly, a larger number of follow-up participants might have provided more reliable information. There were a few reasons why a large a number of participants were lost to follow-up such as; i) many of the women could not take time off work to attend the clinical examination, ii) some did not want to let their children participate in the trial because of the extensive examination program, and iii) the offspring follow-up was not part of the original LiP trial and so the women had not agreed to take part in the LiPO study at the time of their inclusion in the LiP study. We were not permitted to contact these women so we are unable to quantify the reasons. However, the group who were lost to follow-up had significantly higher 2-h OGTT plasma glucose levels. This is a weakness of our data and might account for the negative results, as we would expect this to increase offspring adiposity.

Since no similar large offspring RCT follow-up studies have been conducted, we have no comparator. Recently, a pilot examined weight development from 0–4 years from questionnaires on 72 children (34 with maternal intervention and 38 controls) and found no effect of intervention (advice on physical activity and diet) [29]. However, the mothers had mainly normal weight, making comparisons with our study difficult. In another trial, women with mild gestational diabetes between 24 and 34 weeks gestation were allocated to dietary advice, blood glucose monitoring and insulin therapy if necessary, or to a control group. Despite a reduction in the prevalence of macrosomia at birth, no difference was seen in child BMI Z-score at the age of 4–5 years [27]. Although not detected in our study, maternal obesity has been linked to offspring obesity in several studies. Children born to obese women have double the risk of being overweight at 24 months compared to children born to normal weight women [5]. Maternal BMI is the strongest predictor of offspring BMI as well as increased fat mass [6]. In our study, only 9% of the children of obese women were classified as overweight or obese at the age of 2.8 years. The prevalence of being overweight or obese and the mean birth weight were similar to the general Danish population [4], [30]. Accordingly, we would suggest that the women who participated in our follow-up study represented a group of obese women who were motivated by health-promotion, and that the children benefitted from their motherś voluntary participation in the LiP study, regardless of the randomization. This is supported by the lack of difference between the offspring of mothers participating in the LiP study and the offspring of the normal weight reference group.

In all measures of anthropometry and body composition at the age of 2.8 years, the LiP offspring were comparable to the reference group of children born to normal weight women. Based on observational studies on the effect of pre-pregnancy BMI this was not expected. Furthermore, reference group women, apart from having a lower BMI, were characterized by having a higher level of education and their children were smaller at birth compared to the LiP mothers and children. The reference group attendance was only 29%, which may reflect a selection bias towards highly educated, healthy women. In theory all of these differences should result in leaner children in the reference group compared to the offspring of the obese women. In contrast, GWG was higher in the reference group. This was expected, as lean women tend to gain more weight during pregnancy than obese women. The higher GWG in the reference group could result in higher BMI Z-score in their offspring, but adjusting for GWG in the multiple regression analyses made little change in this outcome. Unfortunately, we had no information on glucose levels during pregnancy in the reference group. Background information on this group was collected retrospectively from an electronic patient registry and from postal questionnaires, and GWG was based on self-reported values. Furthermore, breastfeeding data was based on questionnaires answered two years post partum. Accordingly, the self-reported GWG and breastfeeding data might be the subject of faulty recall. These limitations must be considered when interpreting our data. However, the purpose of this group was to serve as a reference, not to study the effects of GWG, breastfeeding, glucose values during pregnancy, or other potential factors influencing offspring adiposity.

The main strength of our study is the detailed examinations of mother and child dyads from the LiP study in both pregnancy and early childhood. Limitations include the risk of selection bias towards a group from the LiP study who were highly motivated to improve lifestyle irrespective of the randomization. In addition, there was a low attendance rate and differences in data collection between the LiP study and the reference group.

In conclusion, lifestyle intervention in pregnancy did not result in changes in offspring anthropometrics at 2.8 years. DEXA scan was possible in 83.7% of the children consenting to participate in this part of the examination. When comparing offspring of obese women with offspring of normal weight women, all anthropometric measurements were similar. We speculate that obese pregnant women entering a lifestyle intervention RCT regardless of the intervention are highly motivated to focus on healthy lifestyle during pregnancy. This makes it difficult to determine the effects of the randomized lifestyle intervention compared to an unselected control group of obese women. We await results from other pregnancy intervention studies such as the Maternal Obesity Management (MOM) trial [31] which focuses on offspring outcomes.

## Supporting Information

Protocol S1 (DOC)

Checklist S1 (DOC)
