# Supplementary material for: Anthropometrics and Body Composition by Dual Energy X-Ray in Children of Obese Women: A Follow-Up of a Randomized Controlled Trial (the Lifestyle in Pregnancy and Offspring [LiPO] Study)
Source: PLoS One. 2014 Feb 24;9(2):e89590. doi: 10.1371/journal.pone.0089590 (PMC3933548; doi:10.1371/journal.pone.0089590)
Supplement: Protocol S1 — (DOC) [file pone.0089590.s001.doc]

**29/6-2010**

**The effect of lifestyle intervention in obese women during pregnancy Follow-up of offspring in early childhood. -The Lifestyle in Pregnancy and Offspring (LIPO) study**

## Aim

To determine the effect of lifestyle intervention during pregnancy on obesity, markers of metabolic syndrome and bone mineralization of 2.5-3 year old children of obese mothers.

To assess the effect after controlling for maternal and perinatal factors.

## Background

Over weight and obesity are increasing problems worldwide. These conditions lead to an increased risk of a series of severe diseases – such as cardio-vascular disease, diabetes and cancer (1). In many cases obesity is also associated to risk factors such as insulin resistance, hypertension and dyslipidemia – the so called metabolic syndrome. (2). The mechanism behind this is traditionally considered interplay between genetic and environmental factors, but newer studies has suggested, that epigenetic modulation as a result of the intrauterine and postnatal milieu has an independent role (fetal/neonatal programming).

In Denmark 1/3 of all pregnant women are overweight (body mass index (BMI) >= 25 kg/m^2^), and 12 % are obese (BMI >= 30 kg/m^2^) (3), and their children have an increased risk of being born large for gestational age and of developing obesity in childhood, adolescence and adulthood.

Birth weight is often used as a surrogacy for fetal growth and nourishment. About 20 years ago, the English researches Barker and Hales reported that low birth weight predisposed to glucose intolerance, insulin resistance, hypertension and cardiovascular disease decades later in life (4, 5). On this background, they put forth the birth weight hypothesis (”the thrifty phenotype hypothesis”) in which malnourishment in fetal life leads to a programming of energy conserving mechanisms (6). These mechanisms are inappropriate if the individual is exposed to ordinary western lifestyle and increases the risk of for instance Type 2 diabetes. Low birth weight in combination with rapid postnatal growth is especially a problem (7). Many epidemiological human studies as well as animal studies have shown that also high birth weight is associated with Type 2 diabetes and long term risk of obesity, with insulin resistance playing an essential role (8, 9, 10). Furthermore, high birth weight is associated with increased risk of several forms of cancer (11), and type Type 1 diabetes (12).

High birth weight is especially seen if the mother has diabetes, pregestational obesity and/or excessive gestational weight gain. Maternal obesity is an independent risk factor for a series of diseases/adverse outcomes in their offspring: obesity, dyslipidemia, glucose intolerance, high HbA1c and hypertension (10, 13, 14). Excessive gestational weight gain is also associated with increased BMI, blood pressure and adiposity (15).

Adiposity in children can be determined using measures of BMI, skinfolds and abdominal circumference, but also by using DEXA scans.

Pregestational obesity is also associated with low 25-OH-vitD in both mother and child (16). This can have long term consequences as low vitamin D status influences a range of mechanisms including development of organs and the immune system. Studies have thus shown, that supplements of vitamin D given to pregnant women and infants reduces the risk of asthma in childhood (17,18,20) and of Type 1 diabetes in adulthood (19), whereas smaller studies have suggested a negative effect of vitamin D on asthma and atopic dermatitis at the age of 9 (21). Maternal 25-OH-vitD is highly correlated to 25-OH-vitD in the umbilical cord (22, 23,24, 25). And umbilical cord 25-OH-vit D is thus considered a good proxy for fetal 25-OH-vit D status.

Maternal over weight/obesity and gestational weight gain are potentially modifiable risk factors, which in theory can be prevented by securing optimal gestational weight gain. Therefore, it is key to focus on possible interventions, which can limit gestational weight gain.

The present study´s first part (study A) is a follow-up on an ongoing lifestyle intervention study at Odense University Hospital and Aarhus University Hospital, Denmark, the Lifestyle in pregnancy (LiP) study. The project is registered at www.clinicaltrials.gov (26). In the LiP project 360 obese pregnant women (pregestationel BMI >= 30 kg/m^2^) were randomized to lifestyle intervention with diet and exercise guidance or routine control. The aim was to limit the gestational weight gain and thereby limiting pregnancy- and birth complications. The inclusion criteria were: singleton pregnancies (nulli- and multipara) with pregestational BMI >=30 kg/m2, age 18-40 years and normal oral glucose tolerance test (OGTT).

Exclusioncriteria were: multiple pregnancies, chronic disease (for instance asthma, rheumatoid disease, inflammatory bowel disease, diabetes, alcohol- or substance abuse and non-Caucasians).

Results from the RCT are not yet available. Therefore, it is not known whether the RCT will result in significant effects on either maternal or fetal outcomes. Regardless of the results, it is interesting to determine the effect of the intervention on the growth, metabolism and diseases in the offspring in a follow-up study (study A).

In another part of the study, Study B, we wish to compare children of the non-intervention group of pregestational obese mothers to a reference-group of children born to normal weight mothers, born in the same time period. In this part, we will study the effects of pregestational obesity.

**Hypothesis**

**Study A (follow-up of RCT):**

Intervention with diet and exerzice has possitive effects on offspring health at the age of 2.5-3 years in terms of:

1. Obesity (via fetal programming – excess nutrients).
2. Markers of later metabolic syndrome (via fetal programming – excess nutrients).
3. Bone mineralization (via effect of fetal vitamin D status)

**Study B:**

Pregestational obesity effects the offspring at the age of 2.5-3 years in terms of:

1. Obesity (via fetal programming – excess nutrients).
2. Markers of later metabolic syndrome (via fetal programming – excess nutrients).
3. Bone mineralization (via effect of fetal vitamin D status)

**Materials and methods:**

**Study A:**

Design:

Randomized controlled trial. Prospective follow-up.

Inclusion criteria:

Children born to mothers, who participated in the LiP study in Odense University Hospital or Aarhus University Hospital.

Numbers:

Estimated drop-out of the LiP study is 50%. This study will thus contain:

- Children born to obese mothers who participated in lifestyle intervention during pregnancy (n=90)
- Children born to obese mothers who did not participate in lifestyle intervention (n=90)

**Study B:**

Design:

Observational study of 2 groups with the exposition variable +/- pregestationel maternel obesity.

Inclusion criteria:

Children born to mothers who were part of the control group in the LiP study, called the exposed group. Singleton children born to mothers who were normal weight pregestationally (BMI 18,5-24,9 kg/m2), age 18-40 years, normal OGTT and no exclusion criteria from the LiP study, called the reference group.

Exclusion criteria:

Congenital anomalies and chromosome defects.

Numbers:

With an estimated drop-out form the LiP study of 50 % a maximum of 90 children can be included in the exposition group. The reference group must also be 90 children.

***Measures, studies A og B:***

1. 25-OH-Vit D analyses on umbilical cord blood (study A).
2. Questionnaires at 12, 24 and 30-36 months of age. Data: early feeding patterns, breastfeeding patterns etc., exposition to smoking, parental height and weight. The earliest included children will however, not be included in the 12 months questionnaire.
3. Data of early growth- collected from general practitionars.
4. Clinical exam at the age of 30-36 months. Height, weight, abdominal circumference, subscapular and triceps skinfolds and blood pressure.
5. Venous blood sample at the age of 30-36 months: fasting blood glucose, insulin, C-peptide, HbA1c and lipids.
6. DEXA scan for fat mass and mineralization.

**Outcomes:**

1. Studies A og B´s primary outcomes are child BMI SDS (z-score)
2. Secondary outcomes:

a) Abdominal circumference, abdominal circumference:height ratio, subscapular- and triceps-skinfolds, fat percentage by DEXA scan. Bonemineralization by DEXA scan.

b) F-blood glucose, insulin, C-peptide, insulin: glucose ratio, C-peptide: glucose ratio, HOMA index, lipids, 25-OH-vit D and blood pressure for assessment of markers of metabolic syndrome.

**Statistics:**

Power calculation is based on an estimated dropout of 50 % from the LiP study, n = 90 in each group.

BMI SDS:

N in each group is 90, Z_2α_=1.96 (significance level. p=0.05) and Z_2β_=0.84 (strength β=0.80), SD=1, true difference in outcome δ_1_ is X.

This is used in the formula:

n > 2 (((Z_2α_ + Z_2β_ )*SD)/ δ_1_))^2^ (27).

And:

n > 2 (((1.96+0.84)*1)/X))^2^,

which means X= 0.417.

This means, that the study has enough power, if the true difference between (a) intervention group and og non-intervention group of obese pregnant women and between (b) non-intervention group and reference group, is 0,42 SDS – corrected for confounders.

There are no previous studies to base this power calculation on.

**Statistical analyses**

Outcomes will be analyzed according to pregestational BMI *(Study A)* and gestational weight gain *(Study B).* Data will also be analyzed with regression models for controlling of confounders.

**Risks, side effects and inconveniencies**

As part of the study, a clinical exam will be performed and 1 venous blood sample will be made. Except for the discomfort from the blood sample, no side effects are present. A DEXA scan will also be performed. DEXA has a very low level of irradiation and is generally accepted for research purposes in children. Total irradiation dose is 0,001 mSv. For comparison, the background irradiation is 3 mSv - 3000 times larger.

The inconveniences of the study are primarily time consumption for participants. The total exam program is 1.5 hours for each participant.

**Ethics**

The study is conducted according to the Helsinki II-declaration. In both written and oral information it is pointed out that participation is voluntary and that participants at all times can withdraw without any consequences. The Danish data protection agency has approved the study.

Results will be made accessible in international journals and both negative as well as positive results will be reported.

**Informed consent:**

First contact to participants for the clinical exam will be by mailed letter. Participants’ mothers have already participated in the LiP project or/and in a questionnaire survey. Therefore, we already have been in contact with the mothers. The study will be described in writing. If the parents wish to participate or to hear more, they are encouraged to contact Mette Tanvig, MD, PhD student. In that case, they will be invited to oral information. Parents are also advised that they can bring an outsider to the oral information, if they wish. After the information, a period of 2 weeks is given for the parents to make a decision.

**Economy:**

Future PhD student Mette Tanvig has been awarded salary from the University of Southern Denmark and from Region of Southern Denmark. Further funding will be applied for, by for instance NOVO Nordisk Foundation, the Tryg Foundation and the A.P: Møller Mærsk Foundation.

Initiative for the project was made by consultant, associate professor, Ph.D. Dorte Møller Jensen, Department of Endocrinology in close collaboration with MD, future Ph.D. student Mette Tanvig, Ph.D. student Christina Anne Vinter, consultant, associated professor, Ph.D. Jan Stener Jørgensen and consultant, Ph.D. Henrik Thybo Christesen.

**Projectgroup:**

- MD, future PhD. student Mette Tanvig
- MD, Ph.D. student Christina Anne Vinter
- PhD. Jan Stener Jørgensen
- Ph.D. Henrik Thybo Christesen
- Professor Henning Beck-Nielsen
- Consultant, associate professor, Ph.D. Dorte Møller Jensen

**Literature:**

1. Obesity: preventing and managing the global epidemic. Report of a WHO consultation on obesity. WHO Technical Report Series, no. 840, 2000.
2. World Health Organization. Definition, diagnosis and classification of diabetes mellitus and its complications. Report of a WHO consultation 1999.
3. FØDSLER, GRAVIDITET OG BMI 2004 - 1. HALVÅR 2008: Nye tal fra Sundhedsstyrelsen 2008: 9.
4. Hales CN, Barker DJ, Clark PM, Cox LJ, Fall C, Osmond C, Winter PD. Fetal and infant growth and impaired glucose tolerance at age 64. BMJ. 1991 oct 26; 303 (6809):1019-22.
5. Phillips DI, Barker DJ, Hales CN, Hirst S, Osmond C. Thinness at birth and insulin resistance in adult life. Diabetologia. 1994: Feb; 37(2): 150-4.
6. Hales CN, Barker DJ. Type 2 diabetes (non-insulent-dependent) diabetes mellitus: the thrifty phenotype hypothesis. Diabetologia. 1992 Jul: 35(7): 595-601.
7. Yajnik CS, Deshpande SS, Jackson AA, Refsum H, Rao S, Fisher DJ, Bhat DS, Naik SS, Coyaji KJ, Joglekar CV, Joshi N, Lubree HG, Deshpande VU, Rege SS, Fall CH. Vitamin B12 and folate concentrations during pregnancy and insulin resistance in the offspring: the Pune Maternal Nutrition Study. Diabetologia. 2008 Jan;51(1):29-38.
8. Simmons R. Perinatal programming of obesity. Semin perinatol. 2008 Oct; 35(5): 371-4.
9. Gluckman PD, Hanson MA, Cooper C, Thornburg KL. Effect of in uteru and early-life conditions on adult health and disease. N Engl J Med. 2008 Jul 3;359(1):61-73.
10. Boney CM, Verma A, Tucker R, Vohr BR. Metabolic syndrome in childhood: association with birth weight, maternal obesity, and gestational diabetes mellitus. Pediatrics. 2005 Mar;115(3):e290-6.
11. Ahlgren M, Wohlfahrt J, Olsen LW, Sørensen TI, Melbye M.Birth weight and risk of cancer. Cancer. 2007 Jul 15;110(2):412-9.
12. Harder T, Roepke K, Diller N, Stechling Y, Dudenhausen JW, Plagemann A. Birth weight, early weight gain, and subsequent risk of type 1 diabetes: systematic review and meta-analysis. Am J Epidemiol. 2009 Jun 15;169(12):1428-36. Epub 2009 Apr 10. Review.
13. Thomas C, Hyppönen E, Power C. Prenatal exposures and glucose metabolism in adulthood: are effects mediated through birth weight and adiposity? Diabetes Care. 2007 Apr;30(4):918-24. Epub 2007 Feb 2.
14. Aarup M, Sokolowski I, Lous J. The prevalence of obesity and overweight among 3 year-old children in the municipality of Aalborg and identification of risk factors. Ugeskr F læger. 2008 Feb 4;170(6):452-6.
15. Oken E, Taveras EM, Kleinman KP, Rich-Edwards JW, Gillman MW. Gestational weight gain and child adiposity at age 3 years. Am J Obstet Gynecol. 2007 Apr;196(4):322.e1-8.
16. Bodnar LM, Catov JM, Roberts JM, Simhan HN. Prepregnancy obesity predicts poor vitamin D status in mothers and their neonates.J Nutr. 2007 Nov;137(11):2437-42.
17. Litonjua AA, Weiss ST. Is vitamin D deficiency to blame for the asthma epidemic?J Allergy Clin Immunol. 2007 Nov;120(5):1031-5. Epub 2007 Oct 24
18. Holick MF. Sunlight and vitamin D for bone health and prevention of autoimmune diseases, cancers and cardiovascular disease. Am J Clin Nutr. 2004 Dec;80(6 Suppl):1678S-88S. Review.
19. Hyppönen E, Läärä E, Reunanen A, Järvelin MR, Virtanen SM. Intake of vitamin D and risk of type 1 diabetes: a birth-cohort study.Lancet. 2001 Nov 3;358(9292):1500-3
20. Erkkola M, Kaila M, Nwaru BI, Kronberg-Kippilä C, Ahonen S, Nevalainen J, Veijola R, Pekkanen J, Ilonen J, Simell O, Knip M, Virtanen SM. Maternal vitamin D intake during pregnancy is inversely associated with asthma and allergic rhinitis in 5-year-old children.Clin Exp Allergy. 2009 Jun;39(6):875-82.
21. Gale CR, Robinson SM, Harvey NC, Javaid MK, Jiang B, Martyn CN, Godfrey KM, Cooper C; Princess Anne Hospital Study Group. Maternal vitamin D status during pregnancy and child outcomes.Eur J Clin Nutr. 2008 Jan;62(1):68-77. Epub 2007 Feb.
22. Sachan A, Gupta R, Das V, Aqarwal A, Awasthi PK, Bhatia V. High prevalence of vitamin D deficiency among pregnant women and their newborns in northern India. [Am J Clin Nutr.](javascript:AL_get(this, 'jour', 'Am J Clin Nutr.');) 2005 May;81(5):1060-4.
23. Nicolaidou P, Hatzistamatiou Z, Papadopoulou A, Kaleyias J, Floropoulou E, Lagona E, Tsagris V, Costalos C, Antsaklis A. Low vitamin D status in mother-newborn pairs in Greece. Calcif Tissue Int. 2006 Jun;78(6):337-42.
24. Bodnar LM, Catov JM, Roberts JM, Simhan HN. [Prepregnancy obesity predicts poor vitamin D status in mothers and their neonates.](http://www.ncbi.nlm.nih.gov/pubmed/17951482?itool=EntrezSystem2.PEntrez.Pubmed.Pubmed_ResultsPanel.Pubmed_RVDocSum&ordinalpos=12)J Nutr. 2007. Nov ; 137(11):2437-42.
25. Kazemi A, Sharifi F, Jafari N, Mousavinasab N. High prevalence of vitamin D deficiency among pregnant women and their newborns in an Iranian population. J Womens Health (Larchmt). 2009 Jun;18(6):835-9.
26. NTC005304939 ClinicalTrials.gov
27. Armitrage, Berry, Matthews, Statistical Methods in Medical Research, 4^th^ ed, 2002, Blackwell Science Ltd, p. 139
