# Supplementary material for: Anthropometrics and Body Composition by Dual Energy X-Ray in Children of Obese Women: A Follow-Up of a Randomized Controlled Trial (the Lifestyle in Pregnancy and Offspring [LiPO] Study)
Source: PLoS One. 2014 Feb 24;9(2):e89590. doi: 10.1371/journal.pone.0089590 (PMC3933548; doi:10.1371/journal.pone.0089590)
Supplement: Checklist S1 — (DOC) [file pone.0089590.s002.doc]

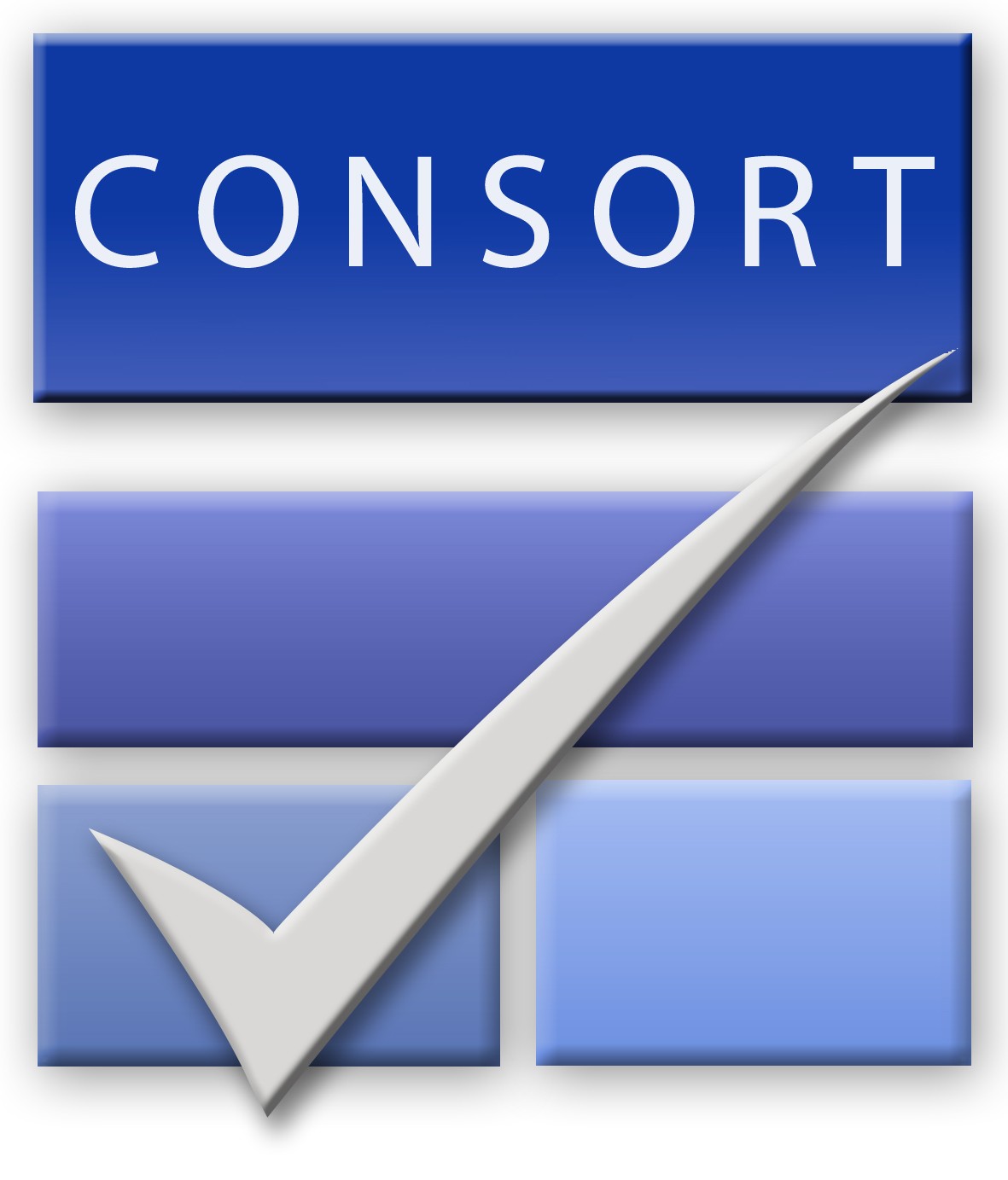
CONSORT 2010 checklist of information to include when reporting a randomised trial*

| Section/Topic | Item No | Checklist item | Reported in Section |
| --- | --- | --- | --- |
| Title and abstract | | | |
|  | 1a | Identification as a randomised trial in the title | Title |
|  | 1b | Structured summary of trial design, methods, results, and conclusions (for specific guidance see CONSORT for abstracts) | Abstract |
| Introduction | | | |
| Background and objectives | 2a | Scientific background and explanation of rationale | Introduction |
|  | 2b | Specific objectives or hypotheses | Introduction |
| Methods | | | |
| Trial design | 3a | Description of trial design (such as parallel, factorial) including allocation ratio | Method section on data collection and handling |
|  | 3b | Important changes to methods after trial commencement (such as eligibility criteria), with reasons | Method section |
| Participants | 4a | Eligibility criteria for participants | Method section on data collection and handling |
|  | 4b | Settings and locations where the data were collected | Method section on data collection and handling |
| Interventions | 5 | The interventions for each group with sufficient details to allow replication, including how and when they were actually administered | Method section on data collection and handling |
| Outcomes | 6a | Completely defined pre-specified primary and secondary outcome measures, including how and when they were assessed | Method section on outcomes |
|  | 6b | Any changes to trial outcomes after the trial commenced, with reasons | Method section on outcomes |
| Sample size | 7a | How sample size was determined | Method section on statistical analyses |
|  | 7b | When applicable, explanation of any interim analyses and stopping guidelines |  |
| Randomisation: |  |  | Method section on data collection and handling |
| Sequence generation | 8a | Method used to generate the random allocation sequence |  |
|  | 8b | Type of randomisation; details of any restriction (such as blocking and block size) | Method section on data collection and handling |
| Allocation concealment mechanism | 9 | Mechanism used to implement the random allocation sequence (such as sequentially numbered containers), describing any steps taken to conceal the sequence until interventions were assigned | Method section on data collection and handling |
| Implementation | 10 | Who generated the random allocation sequence, who enrolled participants, and who assigned participants to interventions | Method section on data collection and handling |
| Blinding | 11a | If done, who was blinded after assignment to interventions (for example, participants, care providers, those assessing outcomes) and how | Method section on data collection and handling |
|  | 11b | If relevant, description of the similarity of interventions |  |
| Statistical methods | 12a | Statistical methods used to compare groups for primary and secondary outcomes | Method section on statistical analyses |
|  | 12b | Methods for additional analyses, such as subgroup analyses and adjusted analyses | Method section on statistical analyses |
| Results | | | |
| Participant flow (a diagram is strongly recommended) | 13a | For each group, the numbers of participants who were randomly assigned, received intended treatment, and were analysed for the primary outcome | Figure 1 |
|  | 13b | For each group, losses and exclusions after randomisation, together with reasons | Figure 1 |
| Recruitment | 14a | Dates defining the periods of recruitment and follow-up | Method section on data collection and handling |
|  | 14b | Why the trial ended or was stopped |  |
| Baseline data | 15 | A table showing baseline demographic and clinical characteristics for each group | Table 1 |
| Numbers analysed | 16 | For each group, number of participants (denominator) included in each analysis and whether the analysis was by original assigned groups | Method section on statistical analyses |
| Outcomes and estimation | 17a | For each primary and secondary outcome, results for each group, and the estimated effect size and its precision (such as 95% confidence interval) | Result section and Table 2 |
|  | 17b | For binary outcomes, presentation of both absolute and relative effect sizes is recommended | Result section and Table 2 |
| Ancillary analyses | 18 | Results of any other analyses performed, including subgroup analyses and adjusted analyses, distinguishing pre-specified from exploratory |  |
| Harms | 19 | All important harms or unintended effects in each group (for specific guidance see CONSORT for harms) | Discussion |
| Discussion | | | |
| Limitations | 20 | Trial limitations, addressing sources of potential bias, imprecision, and, if relevant, multiplicity of analyses | Discussion |
| Generalisability | 21 | Generalisability (external validity, applicability) of the trial findings | Discussion |
| Interpretation | 22 | Interpretation consistent with results, balancing benefits and harms, and considering other relevant evidence | Discussion |
| Other information | | |  |
| Registration | 23 | Registration number and name of trial registry | [NCT00530439](http://care.diabetesjournals.org/external-ref?link_type=CLINTRIALGOV&access_num=NCT00530439), NCT01918319 and NCT01918423  www.[clinicaltrials.gov](http://clinicaltrials.gov/) |
| Protocol | 24 | Where the full trial protocol can be accessed, if available | Protocol S1 |
| Funding | 25 | Sources of funding and other support (such as supply of drugs), role of funders | Section on funding |

*We strongly recommend reading this statement in conjunction with the CONSORT 2010 Explanation and Elaboration for important clarifications on all the items. If relevant, we also recommend reading CONSORT extensions for cluster randomised trials, non-inferiority and equivalence trials, non-pharmacological treatments, herbal interventions, and pragmatic trials. Additional extensions are forthcoming: for those and for up to date references relevant to this checklist, see [www.consort-statement.org](http://www.consort-statement.org/).
